# Full-Band EEG Recordings Using Hybrid AC/DC-Divider Filters

**DOI:** 10.1523/ENEURO.0246-21.2021

**Published:** 2021-08-24

**Authors:** Azat Nasretdinov, Alexander Evstifeev, Daria Vinokurova, Gulshat Burkhanova-Zakirova, Kseniya Chernova, Zoya Churina, Roustem Khazipov

**Affiliations:** 1 Laboratory of Neurobiology, Kazan Federal University, Kazan 420008, Russia; 2 Institut de Neurobiologie de la Méditerranée (INMED), Aix-Marseille University, Institut National de la Santé et de la Recherche Médicale, Marseille 13273, France

**Keywords:** DC recordings, EEG, inverse filter

## Abstract

Full-band DC recordings enable recording of slow electrical brain signals that are severely compromised during conventional AC recordings. However, full-band DC recordings may be limited by the amplifier’s dynamic input range and the loss of small amplitude high-frequency signals. Recently, Neuralynx has proposed full-band recordings with inverse filtering for signal reconstruction based on hybrid AC/DC-divider RRC filters that enable only partial suppression of DC signals. However, the quality of signal reconstruction for biological signals has not yet been assessed. Here, we propose a novel digital inverse filter based on a mathematical model describing RRC filter properties, which provides high computational accuracy and versatility. Second, we propose procedures for the evaluation of the inverse filter coefficients, adapted for each recording channel to minimize the error caused by the deviation of the real values of the RRC filter elements from their nominal values. We demonstrate that this approach enables near 99% reconstruction quality of high-potassium-induced cortical spreading depolarizations (SDs), endothelin-induced ischemic negative ultraslow potentials (NUPs), and whole-cell recordings of membrane potential using RRC filters. The quality of the reconstruction was significantly higher than with the existing inverse filtering procedures. Thus, RRC filters with inverse filtering are optimal for full-band EEG recordings in various applications.

## Visual Abstract

**Figure:**
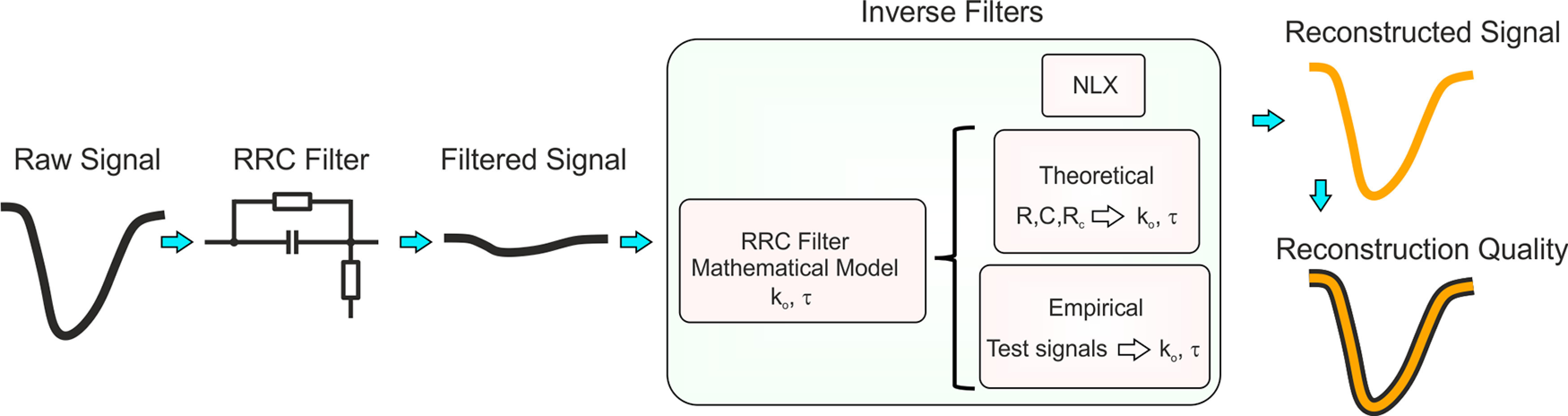

## Significance Statement

This study describes an optimized inverse filtering procedure with calibrated passive filter parameters for high-quality full-band EEG recordings using hybrid AC/DC-divider filters, and shows that this approach provides significantly higher quality reconstruction of cortical spreading depolarizations (SDs), ischemic negative ultraslow potentials (NUPs), and whole-cell recordings of membrane potential than the existing inverse filtering procedure.

## Introduction

While the conventional bandwidth of clinical EEG encompasses frequencies above 0.5 Hz, several important physiological and pathologic patterns of brain activity occur within the infra-slow (<0.5 Hz) frequency range (Kovac et al., 2018). These include retinal waves- driven slow activity transients (Vanhatalo et al., 2005a; Colonnese and Khazipov, 2010), activity associated with different cognitive tasks and behavior (Birbaumer et al., 1990; Cui et al., 2000), resting state networks (Fox and Raichle, 2007; Grooms et al., 2017), and infra-slow oscillations during slow-wave sleep (Vanhatalo et al., 2004; Onton et al., 2016; Lecci et al., 2017; Miyawaki et al., 2017). Also included in infra-slow activity are long, high amplitude DC shifts during focal onset seizures (Voipio et al., 2003; Rodin et al., 2014) and the continuum of spreading depolarizations (SDs) during epilepsy, migraine, brain trauma, and ischemia (for review, see Dreier, 2011; Pietrobon and Moskowitz, 2014; Dreier and Reiffurth, 2015; Herreras and Makarova, 2020). Finally, extremely slow and large SD-initiated negative ultraslow potentials (NUP) have recently been reported in humans during brain ischemia representing the extreme end of the SD continuum (Oliveira-Ferreira et al., 2010; Drenckhahn et al., 2012; Hartings et al., 2017; Carlson et al., 2018; Dreier et al., 2018, 2019; Lückl et al., 2018).

**Figure 1.**
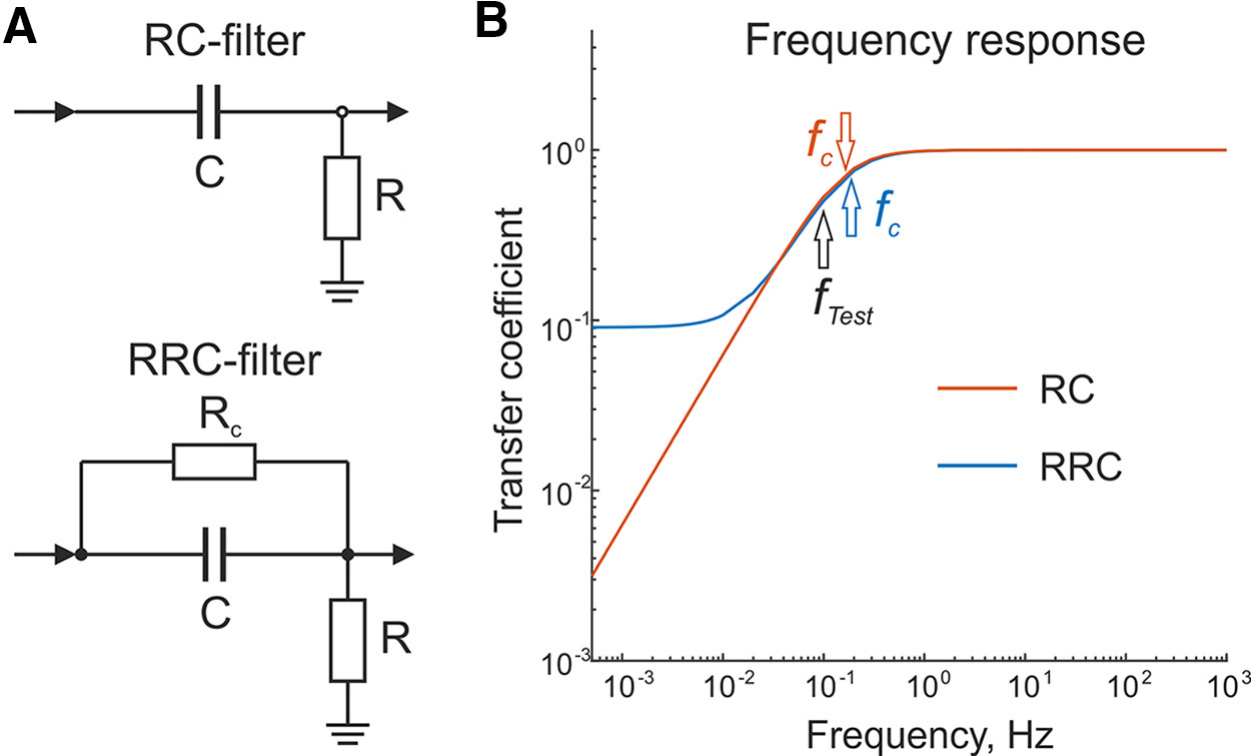
Comparison of RC filter and RRC filter properties. ***A***, Circuit diagrams of an RC filter (top panel) and an RRC filter (bottom panel). ***B***, Frequency responses (in log scale) of the RC filter (in red) and the RRC filter (in blue). Values of the filter elements were: *R *=* *1 MΩ, *R_C_* = 10 MΩ, C = 1 μF. *f_c_* indicates the cutoff frequency for each frequency response, *f_Test_* indicates the frequency (0.1 Hz) of the harmonic signal used in the empirical estimation of the inverse filter coefficients.

Full-band DC recordings are the gold standard for exploration of infra-slow activity (Vanhatalo et al., 2005b; Dreier et al., 2017). However, in keeping with the power-law rule, infra-slow activities typically have larger amplitude than activity in fast frequency bands (Buzsáki and Draguhn, 2004). Along with the common problems of large signal offsets and drifts intrinsic to amplifiers and/or caused by poorly controlled electrochemical processes at the electrodes, full-band DC recordings impose the use of amplifiers with large (hundreds of millivolts) input ranges and high-resolution ADC that increases the cost of equipment tremendously. Alternatively, inverse filtering has been proposed for reconstruction of infra-slow electrophysiological signals from AC recordings (Hartings et al., 2009; Abächerli et al., 2016). The accuracy of reconstruction of ultraslow signals and constant DC signals with this approach is limited, however, because of severe attenuation of the signal along with a reduction in frequency that is an inherent feature of RC filters (Fig. 1*A*,*B*, RC filter). Neuralynx has proposed improved DC signal transfer by using a resistance (DC-divider) introduced in parallel to the capacitor in the RC filter chain as realized in the input filter of Digital Lynx SX Neuralynx amplifiers (Fig. 1*A*,*B*, RRC filter; https://support.neuralynx.com/hc/en-us/articles/360054937932-Hybrid-DC-Coupling-and-Getting-Back-to-Unity-Gain). Digital inverse filtering has further been proposed for reconstruction of full-band signals from recordings obtained using such a hybrid AC/DC-divider filter (Hybrid_Input_compensation_v.1.0, https://neuralynx.com/software/hybrid-input-compensation, hereafter referred to as *IF_NLX_*. However, the quality of signal reconstruction of biological signals has not yet been assessed. Further, the versatility of the NLX routine is limited by the standard set of inverse filter coefficients, which may vary between channels. Here, we propose a digital inverse filter based on a mathematical model describing the RRC filter, which provides high computational accuracy and versatility, and procedures for the evaluation of the inverse filter coefficients, adapted for each recording channel to minimize the error caused by deviation of the real values of the RRC filter elements from the nominal values. We demonstrate that this approach enables near 99% reconstruction quality of high-potassium induced SDs, endothelin-induced ischemic NUPs, and whole-cell recordings of membrane potential using RRC filters.

## Materials and Methods

### Amplitude-frequency characteristics of RC and RRC filters

The rationale for using RRC filters for full-band recordings and reconstructions is illustrated by Figure 1. Commonly used RC filters for AC recordings (Fig. 1*A*, top panel) display sharp signal attenuation below the cutoff frequency *f*_C_, with the transfer coefficient approaching zero values at zero frequency (DC signal; Fig. 1*B*, red). The addition of Rc resistance in parallel to the capacitor C of the RC chain (as realized in the input filter of Neuralynx Digital Lynx SX systems) does not affect the transfer coefficient at frequencies above *f_C_*, causes smoother signal attenuation at frequencies below *f_C_*, and at infra-slow frequencies, the transfer coefficient (*k_0_*) approaches a plateau value defined by $k_{0}=R/(R+R_{C})$ ratio. The latter plateau value corresponds to the transfer coefficient of DC signal. Thus, the RRC filter displays hybrid properties operating as a DC-divider at low frequencies and as a standard AC filter at higher frequencies.

### Reconstruction of full-band signals through inverse filtering

The complex transfer function (K(iω)) for the circuit from Figure 1*A*, bottom panel, is the following:

(1) $$K\left(i\omega \right)=\frac{R+i\omega CR_{C}R}{R_{C}+R+i\omega CR_{C}R},$$

where i is an imaginary unit and ω is an angular frequency.

The frequency response (FR(ω)) of this filter is described as

(2) $$FR\left(\omega \right)=\left|K\left(i\omega \right)\right|=\sqrt{\frac{R^{2}+{\left(\omega CR_{C}R\right)}^{2}}{{\left(R+R_{C}\right)}^{2}+{\left(\omega CR_{C}R\right)}^{2}}}.$$

An inverse filter can be used to reconstruct signals passing through the RRC filter. The reconstruction filter in the Laplace representation for Equation 1 has the following transfer function:

(3) $$K_{rec}\left(s\right)=\frac{1}{K(s)}=\frac{R_{C}+R+sCR_{C}R}{R+sCR_{C}R},$$

where *s* is a complex number frequency parameter.

The filter (Eq. 3) is stable at all frequencies since it has a single negative pole *s_p_* (the value of *s* at which $K_{rec}\left(s\right)$ is equal to infinity) described as $s_{P}=-1/(CR_{C})<0$. In practice it is convenient to use a digital analog of the filter. By applying the bilinear transform (T is sampling interval and $z=e^{sT}$)

(4) $$s=\frac{2}{T}\frac{\left(z-1\right)}{\left(z+1\right)},$$

the z-representation of the reconstruction filter ($K_{rec}\left(z\right)$) can be obtained

(5) $$K_{rec}\left(z\right)=\frac{\left(R_{C}+R\right)T+2CR_{C}R+\left(\left(R_{C}+R\right)T-2CR_{C}R\right)z^{-1}}{RT+2CR_{C}R+(RT-2CR_{C}R)z^{-1}}=\frac{b_{1}+b_{2}z^{-1}}{a_{1}+a_{2}z^{-1}}.$$

Thus, the numerator and denominator of this equation contain coefficients $b_{1},b_{2},a_{1},a_{2}$ used in the digital inverse filter. The bilinear transform was chosen to maintain the stability of the original filter. The error caused by frequency warping introduced by the bilinear transform is insignificant as the filter cutoff frequency is much lower than the sampling frequency. To estimate the frequency response error, we calculated the error function $err\left(f\right)=|FR\left(f\right)-FR(f_{w}\left(f\right))|$, where *f* is frequency f=ω/2π, and $f_{w}\left(f\right)=(1/\pi T)\text{tan}(\pi Tf)$ describes frequency warping of the bilinear transform. The *err* value was <10^−10^ through all frequencies. Hereafter, the bilinear transform error will be ignored.

### Empirical estimation of the coefficients for the inverse filter

**Figure 2.**
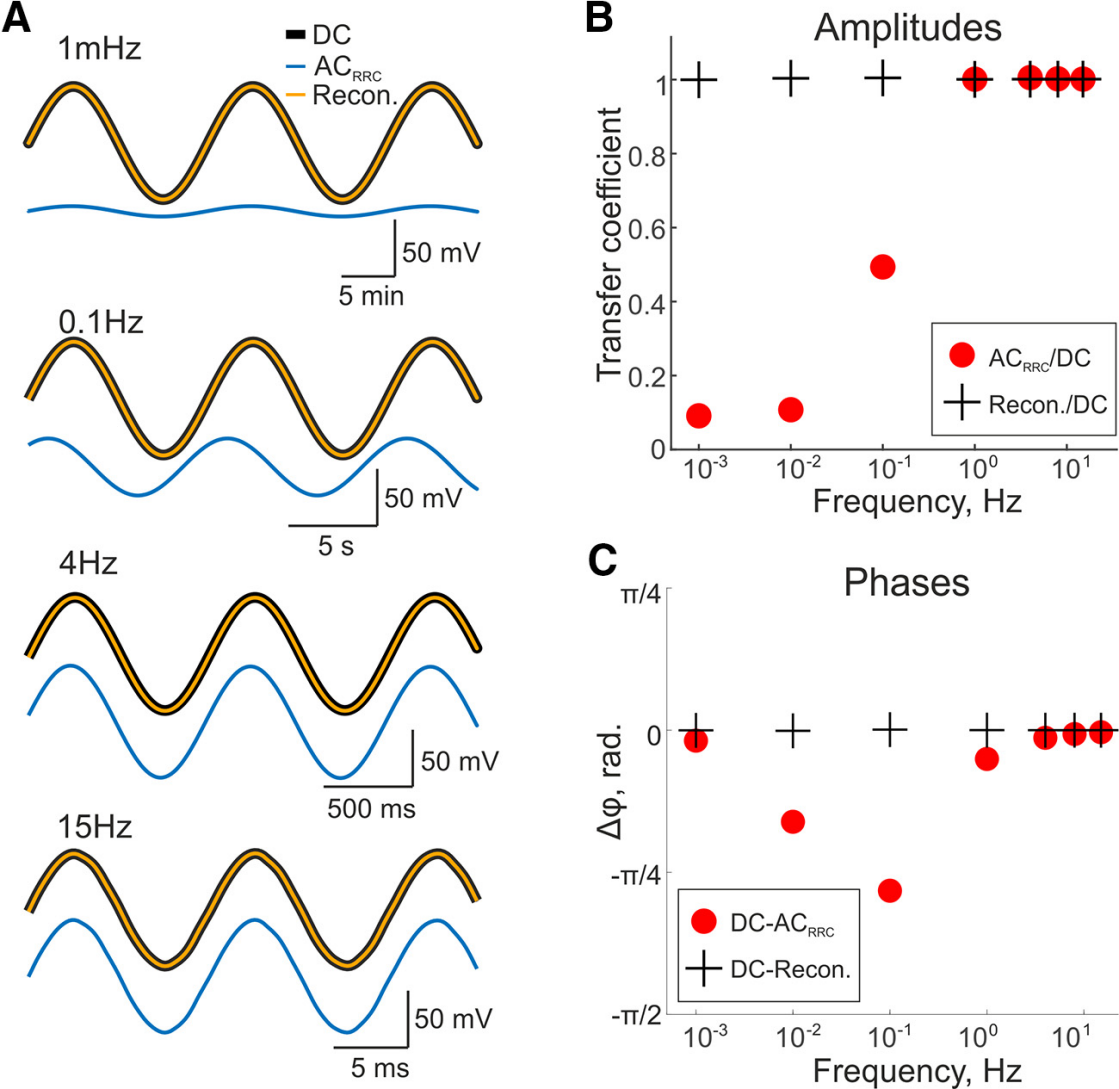
Investigation of the RRC filter characteristics using test sine signals. ***A***, Examples of sine signals at frequencies 1 mHz, 0.1 Hz, 4 Hz, and 15 Hz recorded simultaneously by the AC_RRC_ (blue trace) and DC (black trace) channels as well as the result of signal reconstruction via *IF_EMPIR_* (inverse filter empirical) from AC_RRC_ recording (orange trace). ***B***, Amplitude ratio of sine signal recorded in AC_RRC_ and DC modes at seven selected frequencies: 1 mHz, 10 mHz, 0.1 Hz, 1 Hz, 4 Hz, 8 Hz, and 15 Hz (red circles) and the amplitude ratio of reconstructed signal relative to the original signal recorded in DC mode (black crosses). ***C***, Phase difference between DC and AC_RRC_ signals (red circles) containing sine signals at the same frequencies as shown in panel ***B***, and the phase difference between DC and reconstructed from AC_RRC_ signals (black crosses).

The discrete R and C components of the RRC filter may significantly vary from the nominal values (1–5% is typical) and this is a major concern for reconstruction most critically in terms of phase delay in the middle of the band over which the transfer coefficient is increasing (Fig. 2*C*). Therefore, it is essential to verify these values to ensure reconstruction quality. The parameters from Equation 5 can be assessed through the analysis of responses to specially constructed signals delivered to the amplifier input. This can be done either by the method described in (Hartings et al., 2009) or by the procedure that follows. None of the three parameters (*R*, *R_C_*, *C*) can be measured having only the voltage on time dependencies, but it is possible to determine their dimensionless or time-dependent combinations. By using the notations $k_{0}=R/(R+R_{C})$ and $\tau =CR_{C}$ Equations 2, 5 can be rewritten as

(6) $$K_{rec}\left(z\right)=\frac{\left(T+2k_{0}\tau \right)+(T-2k_{0}\tau )z^{-1}}{\left(Tk_{0}+2k_{0}\tau \right)+(Tk_{0}-2k_{0}\tau )z^{-1}}=\frac{b_{1}+b_{2}z^{-1}}{a_{1}+a_{2}z^{-1}}$$

(7) $$FR(f)=\sqrt{\frac{k_{0}^{2}+{\left(2\pi f\tau k_{0}\right)}^{2}}{1+{\left(2\pi f\tau k_{0}\right)}^{2}}},$$

where

(8) $$k_{0}=FR\left(0\right)=\frac{V_{out1}-V_{out0}}{V_{in}}.$$

*V_out1_* and *V_out0_*, output voltages during two constant levels *V_in_* and 0 with at least 200 s in duration. The duration of the test signal should be selected based on the temporal characteristics of the signal to be reconstructed. This is done to account for the effect of drift over the characteristic time of the signal.

The parameter *τ* defined from Equation 7 as

(9) $$\tau =\frac{1}{2\pi fk_{0}}\sqrt{\frac{k_{f}^{2}-k_{0}^{2}}{1-k_{f}^{2}}},$$

where $k_{f}=FR\left(2\pi f\right)=V_{outh}/V_{inh}$ is measured by applying the harmonic signal *V_inh_* with amplitude 200 mV and frequency f = 0.1 Hz (for the Neuralynx amplifier). This frequency should correspond to the average value between the stopband and passband levels of the frequency response (indicated as *f_Test_* in Fig. 1*B*). To increase the SNR, the amplitudes *V_inh_* and *V_in_* should commensurate with the input range of the amplifier. We recommend generating signals using a DAC with at least 12-bit resolution. During reconstruction, the exponential transient process of the filter adjustment to the signal offset should be taken into account. This can be bypassed by prerecording a signal with a duration of at least 5*τ* (for <1% error) or more, or by *post hoc* addition of estimated values of the offset with the required duration to the start of recorded signal before reconstruction.

### Surgery and recordings from animals

The animal experiments were conducted in compliance with the appropriate Animal Research: Reporting In Vivo Experiments (ARRIVE) guidelines. Animal care and procedures were in accordance with EU Directive 2010/63/EU for animal experiments, and all animal-use protocols were approved by the French National Institute of Health and Medical Research (APAFIS #16992-2020070612319346 v2) and the Local Ethical Committee of Kazan Federal University (No. 24/22.09.2020). Wistar rats of both sexes from postnatal day (P)16 to P60 were used. Intracortical recordings were performed on head restrained urethane-anaesthetized (1.2–1.5 g/kg, i.p.) rats as described previously (Nasretdinov et al., 2017). Recordings were performed using 16-channel linear probes with 100-μm separation distance (Neuronexus Technologies) from the barrel cortex. The probe was inserted to the barrel cortex to a target depth of ∼1600-μm SDs were evoked by distant epipial 1 m KCl application above the prefrontal or visual cortex. Signals from the silicone probe were amplified (1000×) and recorded using a Digital Lynx SX amplifier (Neuralynx) in DC mode after offset compensation (Nasretdinov et al., 2017). SD-initiated NUPs were recorded during 1-h-long local epipial application of the vasoconstrictor endothelin-1 [ET-1; Sigma; 1 μm solved in artificial CSF (ACSF)] followed by 1 h of wash with ACSF (Dreier et al., 2002; Oliveira-Ferreira et al., 2010). Patch-clamp recordings were obtained from layer 5 (L5) neurons of the barrel cortex *in vivo* with borosilicate glass patch pipettes of 5- to 7-MOhm resistance when filled with solution of the following composition: 144 mm K-gluconate, 4 mm KCl, 4 mm Mg-ATP, 10 mm Na_2_ phosphocreatine, 0.3 mm Na GTP, and 10 mm HEPES (pH 7.3). Whole-cell signals were recorded using an Axopatch 200B amplifier and acquired using a Digidata 1440A (Molecular Devices), and/or Digital Lynx SX (Neuralynx). All recorded signals were replayed using the Multiclamp 1440A built-in DAC and acquired using a Digital Lynx SX.

### Data analysis

Data analysis was performed using custom-written and built-in functions in MATLAB (The MathWorks). Amplitude ratios and phase shifts of sinusoidal signals were estimated using fast-Fourier transform. Signal amplitudes were calculated as the most negative value of LFP after baseline subtraction. Slopes of the signals were calculated from the first LFP derivate. The values in the text are presented as mean value ± standard deviation. To estimate the quality of reconstruction the percentage root mean square difference (PRMSD) was calculated as

(10) $$\%PRMSD=100\%\cdot \sqrt{\frac{\sum_{n=1}^{N}{\left(V_{DC}-V_{rec}\right)}^{2}}{\sum_{n=1}^{N}V_{DC}^{2}}}.$$

For ideal reconstruction, PRMSD = 0.

The MATLAB code used for reconstruction is available as Extended Data 1 and online at https://github.com/Nasazat/Inverse-filter.

10.1523/ENEURO.0246-21.2021.ed1 Extended Data 1 MATLAB codes used for the reconstruction: IFtheor.m – reconstruction using *IF_THEOR_*. IFempir.m – reconstruction using *IF_EMPIR_*. Download Extended Data 1, ZIP file.

For SD reconstruction, the analysis intervals included a short period of control before the SD, the SD itself, and a short fragment after it, in total 100 s for each SD. Data were down-sampled to 100 Hz. SDs from all recordings alternating with zero-containing 30-s periods were merged into one long signal to estimate DAC and ADC offset fluctuations. Also, 3 min of zero-valued signal was added to the beginning of the dataset to eliminate the transient process of the filter.

For SD-initiated NUP reconstruction, each recording consisted of a control period, 1 h of ET-1 application and at least 1 h of ET-1 washout. Data were down-sampled to 10 Hz; 5 min of zero-valued signal was added to the beginning of the dataset to eliminate the transient process of the filter. Hundred-second zero intervals have been also added in between recordings from individual animals.

For whole-cell reconstruction 30-s periods of recordings at 32-kHz sampling frequency were used. At the beginning of each episode, 8 s of zero signal were added.

## Results

### Theoretical and empirical estimation of the inverse filter coefficients

The mathematical model of the inverse filter assumes that adequate signal reconstruction depends critically on the value of the two coefficients *k_0_* and *τ* (Eqs. 8, 9). The values of these coefficients can be determined theoretically based on the nominal values of the passive filter parameters according to the equations $k_{0}=R/(R+R_{C})$ and $\tau =CR_{C}$. For the Neuralynx RRC filter with values *R *=* *1 MΩ, *C *=* *1 μF and *R_C_*=10 MΩ, *τ* and *k_0_* are equal to 10 s and 0.0909, respectively (inverse filter with theoretical coefficients, *IF_THEOR_*). However, the actual values of the filter elements may differ from the nominal values, which should lead to distortions when using an inverse filter. Therefore, we conducted an experimental estimate of *τ* and *k_0_* (inverse filter with coefficients measured empirically, *IF_EMPIR_*) for each channel of the amplifier. First, a constant voltage of 1 V (*V_in_*) was applied. This value was selected in accordance with the value *k_0_* = 1/11 to provide a high SNR and to stay within the input range of the amplifier (±131 mV). To estimate the offset, a constant voltage of 0 V was also applied. After ∼2 min of prerecording (corresponds to error <0.001%), the resulting AC_RRC_ recordings were averaged over the flat 200-s intervals and the corresponding *V_out1_* and *V_out0_* values were estimated. The duration of interval for the average (200 s) was selected to minimize the contribution of drift and to provide acceptable reconstruction quality for both SD-initiated NUPs and SDs. Second, a sinusoidal signal of amplitude 200 mV (*V_inh_*, to provide a high SNR and to stay within the input range) and frequency 0.1 Hz (Fig. 1*B*) was applied. The amplitude of the recorded AC_RRC_ signal (*V_outh_*) was estimated using fast-Fourier transform. Coefficients *k_0_* and *τ* were calculated according to Equations 8, 9, respectively. The experimentally measured values of *τ* for 128 channels of the amplifier varied in the range 9.688–10.650 s (average: 10.087 ± 0.204 s), with a deviation from the theoretical value of 10 s by 0.02–6.5% (on average, 1.910 ± 1.121%; *n* = 128). Similarly, the experimentally measured values of *k_0_* varied in the range 0.0904–0.0922 (average: 0.0914 ± 0.0003), with a deviation from the theoretical value of 0.0909 by 0.03–1.38% (on average, 0.54 ± 0.33%; *n* = 128). For all subsequent signal reconstructions using an inverse filter, we used the experimentally determined values of *τ* and *k_0_*. It should be noted that the proposed experimental technique for evaluating the coefficients of an inverse filter is universal and allows one to evaluate these parameters even in the absence of knowledge of the values of the elements of the RRC filter.

### Sine waves

Further, we tested the filter on a series of sinusoidal signals with an amplitude of 50 mV. To estimate amplitude and phase reconstruction we used 5 periods for each frequency (seven values) with total signal duration ∼1.5 h. Signals with different frequencies generated by the DAC were sent simultaneously to the AC_RRC_ and DC channels of the amplifier (Fig. 2*A*).

According to the frequency response (Fig. 1*B*) the RRC filter reduced the amplitudes of sine signals at frequencies of 1 and 10 mHz by 10- to 11-fold. The amplitude of the signal at a frequency of 0.1 Hz decreased by half, while amplitudes of signals with frequencies of 1–15 Hz passed unchanged, although a slight phase shift is still observed for frequencies of 1, 4, and 8 Hz. However, the amplitudes and phases of reconstructed signals did not differ from DC recordings at all frequencies investigated (Fig. 2*B*,*C*).

### SDs

**Figure 3.**
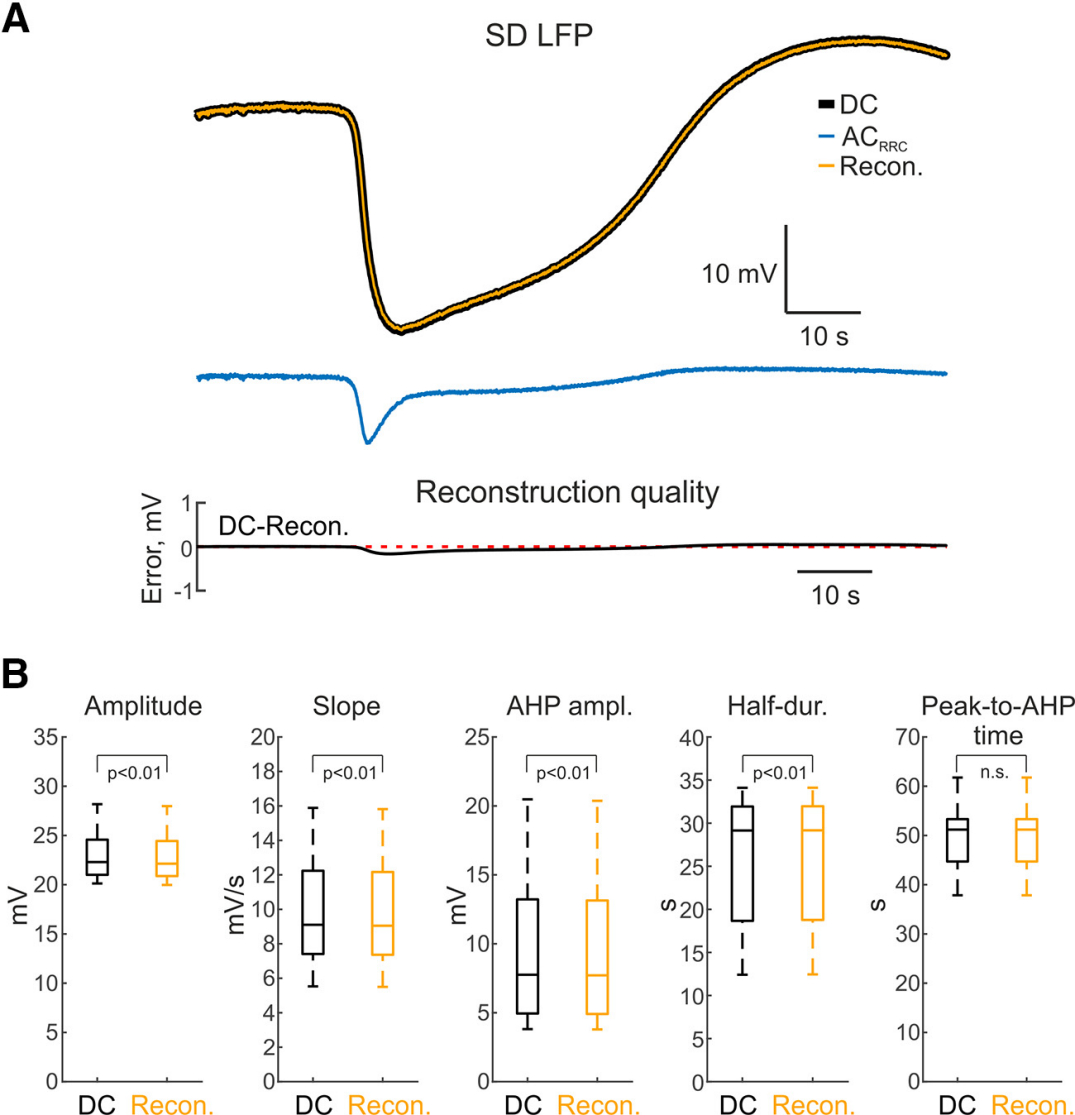
Reconstruction of SD (spreading depolarization) from AC_RRC_ data. ***A***, top panel, Example of SD recorded in DC mode (black), AC_RRC_ mode (blue) and the corresponding DC reconstruction using *IF_EMPIR_* (inverse filter empirical) (orange). Bottom panel, Corresponding reconstruction quality (difference between DC and reconstructed signals), red dashed line behind the trace indicates zero value. ***B***, Boxplots showing SD amplitude, SD slope, AHP (afterhyperpolarization) amplitude, SD half-duration, and negative SD peak to AHP time after reconstruction compared with the corresponding parameters in DC recordings. n.s. - non-significant difference.

At the next stage, we investigated the filter with real SD waveforms. SDs were induced by epipial high-potassium solution application at a distance of 4.2 ± 1.1 mm from a recorded site in the barrel cortex. SDs appeared firstly in the superficial channels and propagated into the deep layers in keeping with previous studies (Nasretdinov et al., 2017; Zakharov et al., 2019). SDs recorded at a depth of 430 ± 110 μm (L2/3) were used for analysis. DC reconstructions were tested on an SD-containing dataset replayed by DAC and recorded by the Neuralynx amplifier simultaneously at two different channels in the DC and AC_RRC_ modes (Fig. 3). Method verification was performed by comparing real-waveform reconstructed signals to signals recorded in DC mode. An example of SD reconstruction is shown on Figure 3*A*. The error of reconstruction (PRMSD) was 1.11 ± 0.11% for *IF_THEOR_* and 0.51 ± 0.05% for *IF_EMPIR_* (*n* = 9 SDs from 9 animals, *p* < 0.05, Wilcoxon signed-rank test). SD reconstruction error was significantly higher than when using *IF_NLX_* (8.55 ± 0.07%; *n* = 9, *p* < 0.01, Wilcoxon signed-rank test). After DC reconstruction, SD amplitude returned to 23.0 ± 2.6 mV, only 0.7 ± 0.1% smaller than the original DC-signal amplitude which was 23.1 ± 2.6 mV (*n* = 9, *p* < 0.01, Wilcoxon signed-rank test; Fig. 3*B*). The slope of the reconstructed signal was also slightly less compared with DC signal (10.1 ± 3.1 mV/s for reconstructed signal 10.2 ± 3.1 mV/s mV for DC, *n* = 9, *p* < 0.01, Wilcoxon signed-rank test; Fig. 3*B*). Afterhyperpolarization (AHP) peak amplitude of SD also had minor differences (10.1 ± 5.6 mV for DC signal and 10.1 ± 5.5 mV for reconstructed, *n* = 9, *p* < 0.01, Wilcoxon signed-rank test; Fig. 3*B*). Timing characteristics of the signals were also comparable, half-duration of SD (duration at the half-amplitude) was 25.7 ± 7.9 s for the DC signal and 25.7 ± 7.9 s for reconstructed (*n* = 9, *p* < 0.01, Wilcoxon signed-rank test; Fig. 3*B*). Time from SD negative peak to AHP peak was 50.0 ± 8.1 s for the DC signal and 50.0 ± 8.1 s for the reconstructed signal (*n* = 9, *p* > 0.05, Wilcoxon signed-rank test; Fig. 3*B*).

### NUPs

**Figure 4.**
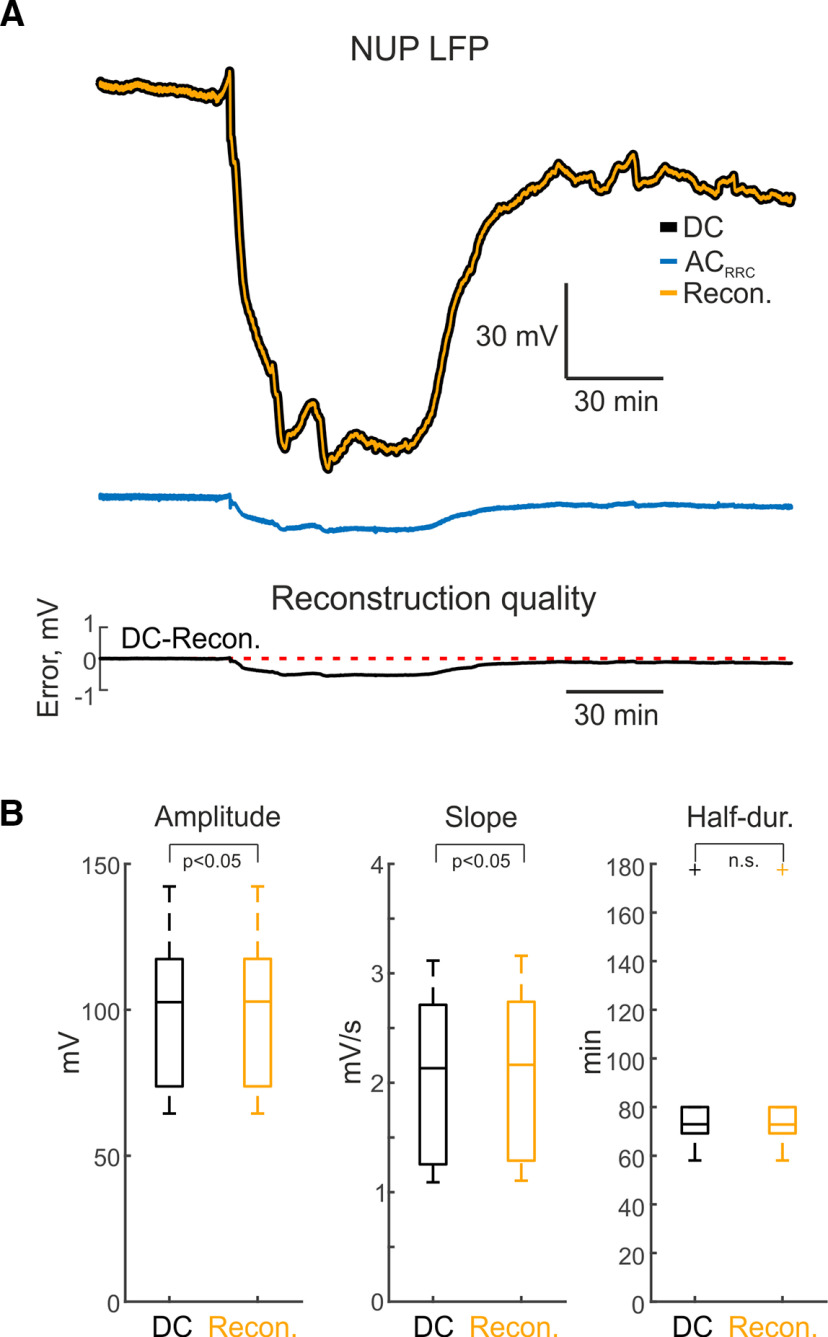
Reconstruction of SD (spreading depolarization)- initiated NUPs (negative ultraslow potentials) from AC_RRC_ data. ***A***, top panel, Examples of SD-initiated NUP recorded in DC mode (black), AC_RRC_ mode (blue) and the corresponding reconstruction using *IF_EMPIR_* (inverse filter empirical) (orange). Bottom panel, Corresponding reconstruction quality (difference between DC and reconstructed signals), red dashed line behind the trace indicates zero value. ***B***, Boxplots showing SD-initiated NUP amplitude, SD-initiated NUP slope and SD-initiated NUP half-duration after reconstruction compared with corresponding parameters in DC recordings. n.s. - non-significant difference.

We next investigated the possibility of this method for reconstruction of an even slower class of signals, ischemic SD-initiated NUPs (Lückl et al., 2018; Dreier et al., 2019). SD-initiated NUPs were evoked by epipial application of the powerful vasoconstrictor ET-1 (1 μm) for 1 h, followed by wash of ET-1 for another 1–3 h (Fig. 4). SD-initiated NUPs progressively developed during 1 h of ET-1 application attaining negative values of −100.5 ± 28.7 mV (at a cortical depth of 530 ± 320 μm, *n* = 6 rats), and decayed on washout of ET-1. Example SD-initiated NUP traces obtained during recordings in DC mode, after passage through the RRC filter and the result of reconstruction using the inverse filter are presented in Figure 4*A*. It is noticeable that RRC filtering profoundly suppressed SD-initiated NUP, but the reconstructed signal fairly matched the original DC-trace. Estimation of reconstruction quality as described above for SD reconstructions revealed that the error did not exceed 1% through the entire course of recordings (Fig. 4*A*, bottom plot), and the PRSMD attained 0.20 ± 0.12% for *IF_THEOR_* and 0.52 ± 0.16% for *IF_EMPIR_* (*n* = 6 SD-initiated NUPs from 6 animals). SD-initiated NUP reconstruction error was significantly higher than that when using *IF_NLX_* (9.07 ± 0.15%; *n* = 6, *p* < 0.05, Wilcoxon signed-rank test). SD-initiated NUP amplitude values after reconstruction using *IF_EMPIR_* matched with the DC signal values (100.6 ± 28.7 and 100.5 ± 28.7 mV, respectively; *n* = 6, *p* < 0.05; Fig. 4*B*). The maximal slope of reconstructed SD-initiated NUP had minimal difference from original DC–recordings (2.1 ± 0.8 and 2.1 ± 0.8 mV/s, respectively, *n* = 6, *p* < 0.05, Wilcoxon signed-rank test; Fig. 4*B*). The half-duration of reconstructed SD-initiated NUP was also similar compared with the DC signal (88.4 ± 44.3 and 88.4 ± 44.3 min, *n* = 6, *p* > 0.05, Wilcoxon signed-rank test; Fig. 4*B*).

### Whole-cell recordings

**Figure 5.**
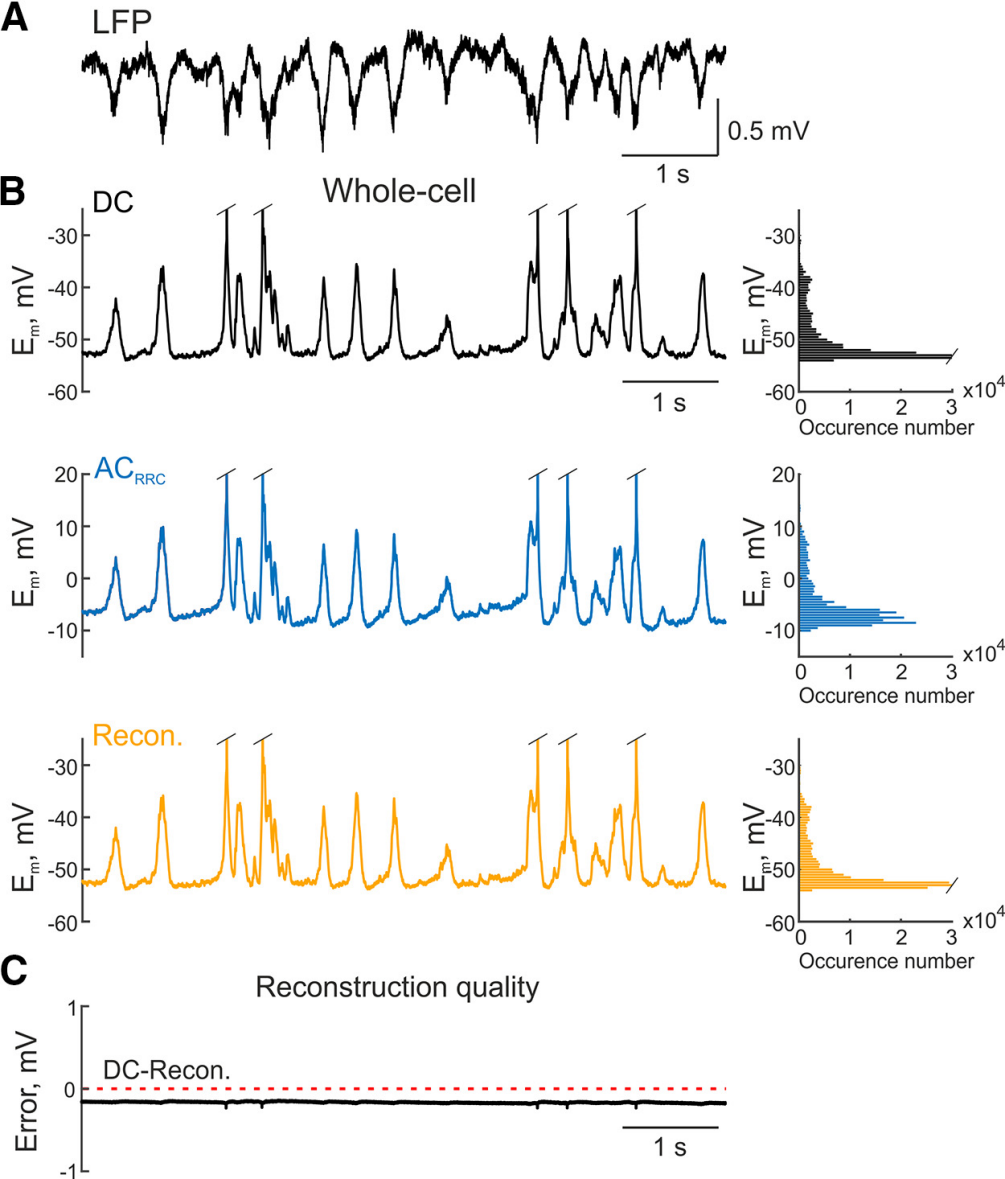
Reconstruction of the membrane potential from whole-cell recordings. ***A***, Example of LFP (local field potential) recording from the rat L5 barrel cortex with several up-down states. ***B***, Example of whole-cell current-clamp DC recording (black trace) from an L5 cell near the LFP recording site shown in ***A***; the same episode recorded in AC_RRC_ mode (blue trace); the result of signal reconstruction from AC_RRC_ data using *IF_EMPIR_* (inverse filter empirical) (orange trace). Histograms on the right show distributions of membrane potential values for each example, respectively. ***C***, Trace demonstrating the difference between the original (DC) and reconstructed membrane potential recordings presented in ***B***, red dashed line indicates zero value.

We also estimated the possibility of reconstructing constant or very slowly changing shifts using recordings of membrane potential from single cells. For this purpose, a recording from an L5 barrel cortex cell was sent simultaneously to the DC and AC_RRC_ channels of the amplifier. Acquiring this data in AC_RRC_ mode can be helpful, for example, for simultaneous recording with LFP (Fig. 5). Slow wave activity was characterized by LFP oscillations with a dominant frequency of 1.9 Hz. This activity at the cellular level had a bimodal pattern with membrane potential fluctuations between hyperpolarized and depolarized states with an average value of −53.4 ± 5.4 mV in DC recordings and −53.7 ± 5.4 mV after reconstruction (*p* < 0.05, Wilcoxon signed-rank test, *n* = 6).

For these recordings we also obtained a high-quality reconstruction with PRMSD of 0.56 ± 0.02% for *IF_THEOR_*, 0.19 ± 0.02% for *IF_EMPIR_*, and 8.64 ± 0.02% for *IF_NLX_*.

## Discussion

In the present study, we developed a procedure for full-band, high quality reconstruction of signals recorded using hybrid AC/DC-divider RRC filters by virtue of inverse digital filtering based on a mathematical model of RRC filters. Our procedure also involves calibration of individual channels to minimize the error caused by deviation of the elements of RRC filters from their nominal values. Through validation in a number of datasets including extracellular recordings of high-potassium-induced cortical SDs, endothelin-induced ischemic NUPs and whole-cell recordings of the membrane potential we demonstrate that this approach enables near 99% reconstruction quality of the original signal in full-band that is superior to the *IF_NLX_* routine which provides reconstruction quality with ∼10% error from the original signal. Our results thus demonstrate that the RRC recording filters proposed by Neuralynx in combination with the inverse filtering routines described here provide high fidelity full-band recordings. This involves not only recordings of infra-slow activity (SDs and SD-initiated NUPs) that can be achieved by reconstructions from AC recordings (Hartings et al., 2009), but also true DC recordings which are a priori impossible using AC recordings with RC input filters. However, several limitations of this approach for full-band recordings remain. These include the internal instrumental DC drift in the electrodes and amplifier. Also, while the approach for full-band recordings described in the present study may be useful for recordings large-amplitude infra-slow signals such as SDs and SD-initiated NUPs, it may be less suitable for recordings of low-amplitude infra-slow activities as division of signal in the infra-slow frequency range will reduce their amplitude and thus decrease SNR for these signals. A potential application for this inverse filtering method could be long-term telemetrical monitoring in animals because the current devices do not provide satisfying solutions for DC recordings. To summarize, our study strongly supports the approach developed by Neuralynx for full-band recordings using hybrid AC/DC-divider filters for exploration of infra-slow activities by providing inexpensive and true DC recordings of large amplitude infra-slow brain signals at no cost to the resolution of the high-frequency activity.

## Synthesis

Reviewing Editor: William Stacey, University of Michigan Decisions are customarily a result of the Reviewing Editor and the peer reviewers coming together and discussing their recommendations until a consensus is reached. When revisions are invited, a fact-based synthesis statement explaining their decision and outlining what is needed to prepare a revision will be listed below. The following reviewer(s) agreed to reveal their identity: Bruce Gluckman.

From the abstract it appeared this was almost a simple advertisement for Neuralynx and their inversion code. But with careful reading it appears you have done much better than their code. They dont address the issue that discrete passive electrical components have significant variability in values (1-5%). Instead, you present a replacement for their inversion filters AND the prescription for calibrating these values for each channel of an amplifier. This is a very important method. Unfortunately, this is not at all clear until the middle of the Results section, and then it is presented in confusing almost hidden way. This needs to be highlighted in the abstract, stated that the derivation of inverse and the methods outlined in the methods section in the introduction, and a short pseudo-code-like protocol stated as a summary in the methods. Likewise your discussion should be reorganized to not be so circular. We have included several suggestions for rewording below. Overall, the point is to be very direct about what you did that was novel, and make it straightforward for others to reproduce these results.

Sharing code:

You mention code and analysis to be available through a GIT repository - but that was not provided. This needs to be made available.

Overall Focus:

It is not until the SD reconstruction section that it becomes clear that this method is somewhat different than the Neuralynx provided inverse filtering. It should be more clear in the abstract, methods, discussion, and introduction that you have made a customized algorithm and how that is novel. A major part of this work is to highlight and present the method of making customized (to each amplifier channel) an inverse filter - and one that is better than the NXL-provided one. This should be the highlight of the paper.

Then the results should start with - prior to all experiments, the inverse filter parameters were calibrated to obtain the parameters ... described in equations XXX YYY.

Clarifications:

Recording Information:

- were whole cell recordings also done from intact animals, or from slices. If latter, then specify preparation

- L5 - specify Layer 5 (L5) cortical neurons (and then specify from where in cortex).

Results:

Sin waves - lowest frequency characterized was 1mHz. To estimate phase reconstruction probably needed ∼10 periods, or ∼3 hours data? Please identify data length. [Note that this is a matter of providing to your reader instructions for properly reproducing this work. ]

SD Reconstruction:

1) if this was all done with previously recorded and published data, then remove the animal details from the methods, and just cite the previously recorded data and paper.

2) Fig 3a - the bottom trace (‘Error mV’) is difficult to read/interpret - provide at least the zero line behind the data. It is hard to determine how close/fast this came back to baseline.

3) The method used here involves taking digitally recorded signals and replaying them. What DAC was used? [Was the output impedance of the DAC and/or filtering of cabling responsible for differences?]

4) It is very confusing to list 3 things that almost sound the same - nominal values, indirect values and NLX routine. Consider different names.

NUP results

by definition NUP data is looking well-below the roll off to the DC, so reconstruction of that signal - with no phase dispersion - is completely expected. Should be noted that this makes sense especially for this reason.

Whole-cell recordings

Please rephrase the following sentence that is hard to understand: “However, the quality of reconstruction is sensitive to fluctuations of internal DC i.e., shift of the amplifier or potential difference on electrodes, which is especially important in long recordings.” The confusion is that aren’t these fluctuations in DC part of the electrical data - and reflect elements of the experiment - not of the reconstruction? Fix the sentence - these are critical to understanding the experiment and should be recorded and analyzed for consistency.

The sentence “Technological scatter of the filter elements values...” is backwards and somewhat ambiguous. The fact that discrete R and C components have significant variation (1-5% is typical) is a major concern for reconstruction - and that reconstruction is most critical in terms of phase delay, and will be most variable in the middle of the band over which the transfer coefficient is increasing (with increasing frequency). We suggest being more explicit to refer to the exact problem, rather than the term “technological scatter of values”.

The phrase “equidistant from the frequency response saturation sections” in the parenthetical comment following equation 8 refers to something most readers wouldn’t be able to follow. Again, please be explicit about the problem here.

The following sentence is somewhat problematic: “Also included in infra-slow activity are long, high amplitude DC shifts during focal onset seizures (Rodin et al., 2014; Voipio et al., 2003)...” We suggest to write the following: “Also included in infra-slow activity are long DC shifts during focal onset seizures (Rodin et al., 2014; Voipio et al., 2003) and the continuum of spreading depolarizations (SD). The latter represent waves of near-complete breakdown of the transmembrane ion gradients associated with neuronal water influx. SDs may occur between epileptic seizures during acute status epilepticus both in animals (Bragin et al., 1997; Kramer et al., 2017; Tamim et al., 2021; Zakharov et al., 2019; Avoli et al., 1991; Hablitz and Heinemann, 1989; Mody et al., 1987) and humans (Dreier et al., 2012; Fabricius et al., 2008; Revankar et al., 2017). Hybrid phenomena between SDs and electrographic seizures in the form of so-called spreading convulsions are rare (Dreier et al., 2012; van Harreveld and Stamm, 1953). As a rule, both SDs and electrographic seizures can be distinguished from each other easily, which is not least related to the fact that the DC shift of SDs is significantly larger than the DC shift of electrographic seizures both in animals (Somjen, 2001) and humans (Revankar et al., 2017). A single short-lasting SD in electrically active tissue is assumed to cause the patient percept of migraine aura because the SD wave can lead in its front to a brief increase and then to a spreading depression of the spontaneous neuronal activity (Ayata and Lauritzen, 2015; Dreier and Reiffurth, 2015; Herreras and Makarova, 2020; Lauritzen et al., 2011; Pietrobon and Moskowitz, 2014;(Hadjikhani et al., 2001; Major et al., 2020; Olesen et al., 1981)). The SD continuum also occurs during brain trauma (Balanca et al., 2017; Bouley et al., 2019; Hartings et al., 2020; Pacheco et al., 2019) and ischemia (Dreier, 2011; Dreier and Reiffurth, 2015; Somjen, 2001)(Dohmen et al., 2008; Dreier et al., 2006; Woitzik et al., 2013).

The last sentence of that paragraph should also be modified somewhat: “Finally, extremely slow and large negative ultraslow potentials (NUPs) with have been recently reported during brain ischemia...” We suggest to write: “Finally, extremely slow and large SD-initiated negative ultraslow potentials (NUP) have been recently reported in humans during brain ischemia representing the extreme end of the SD continuum...”

Throughout the paper, we recommend to write “SD-initiated NUP” instead of simply “NUP” at strategical points in the paper to clarify the connection.

Some interesting points that you might consider adding to the paper:

1. Acute status epilepticus is very different from epilepsy. Status epilepticus induced in animals can trigger a long-lasting plastic process that ultimately leads to the development of chronic epilepsy. Usually, it takes at least 2 weeks before epilepsy has developed out of an acute status epilepticus. The process that leads to epilepsy is termed epileptogenesis, and one of its key features in many epilepsy models is a strikingly selective loss of certain neuron types. Interestingly, in the course of epileptogenesis, the propensity to SD may, however, decline while the propensity to spontaneous seizures increases (Revankar et al., 2017). Thus, the potassium threshold for SD was increased in neocortical slices both from patients who had undergone surgery for intractable epilepsy, and also from rats that had chronic epilepsy following pilocarpine-induced status epilepticus (Maslarova et al., 2011). By contrast, brain slices from age-matched healthy control rats showed a significantly lower threshold. In a similar fashion, the propensity to SD was reduced following epileptogenesis in the course of blood-brain barrier disruption and pentylenetetrazol kindling in rats (Koroleva et al., 1993; Tomkins et al., 2007). Speculatively, the decreased propensity to SD in chronically epileptic tissue could result from the decline in neuron density (Lehmenkuhler et al., 1993) or from upregulation of yet unknown defense mechanisms against SDs.

2. Another class of important DC shifts that may be positive or negative in the human brain and the brains of animals are related to the blood-brain barrier (Kang et al., 2013; Lehmenkühler et al., 1999; Voipio et al., 2003). These DC shifts are physiological rather than pathological. They could also be mentioned briefly because they represent potential confounders in neuromonitoring of electrographic seizures and SDs.

In the methods, the following papers should be cited after “NUPs were recorded during 1 hour long local epipial application of the vasoconstrictor endothelin-1 (ET-1, Sigma, USA; 1 μM solved in ACSF) followed by one hour of wash with ACSF.”: (Dreier et al., 2002; Oliveira-Ferreira et al., 2010)

Avoli M, Drapeau C, Louvel J, Pumain R, Olivier A, Villemure JG. Epileptiform activity induced by low extracellular magnesium in the human cortex maintained in vitro. Ann Neurol 1991; 30: 589-96.

Balanca B, Meiller A, Bezin L, Dreier JP, Marinesco S, Lieutaud T. Altered hypermetabolic response to cortical spreading depolarizations after traumatic brain injury in rats. J Cereb Blood Flow Metab 2017; 37: 1670-86.

Bouley J, Chung DY, Ayata C, Brown RH, Jr., Henninger N. Cortical Spreading Depression Denotes Concussion Injury. J Neurotrauma 2019; 36: 1008-17.

Dohmen C, Sakowitz OW, Fabricius M, Bosche B, Reithmeier T, Ernestus RI, et al. Spreading depolarizations occur in human ischemic stroke with high incidence. Ann Neurol 2008; 63: 720-8.

Dreier JP, Kleeberg J, Petzold G, Priller J, Windmuller O, Orzechowski HD, et al. Endothelin-1 potently induces Leao’s cortical spreading depression in vivo in the rat: a model for an endothelial trigger of migrainous aura? Brain 2002; 125: 102-12.

Dreier JP, Woitzik J, Fabricius M, Bhatia R, Major S, Drenckhahn C, et al. Delayed ischaemic neurological deficits after subarachnoid haemorrhage are associated with clusters of spreading depolarizations. Brain 2006; 129: 3224-37.

Dreier JP, Major S, Pannek HW, Woitzik J, Scheel M, Wiesenthal D, et al. Spreading convulsions, spreading depolarization and epileptogenesis in human cerebral cortex. Brain 2012; 135: 259-75.

Fabricius M, Fuhr S, Willumsen L, Dreier JP, Bhatia R, Boutelle MG, et al. Association of seizures with cortical spreading depression and peri-infarct depolarisations in the acutely injured human brain. Clin Neurophysiol 2008; 119: 1973-84.

Hablitz JJ, Heinemann U. Alterations in the microenvironment during spreading depression associated with epileptiform activity in the immature neocortex. Brain Res Dev Brain Res 1989; 46: 243-52.

Hadjikhani N, Sanchez Del Rio M, Wu O, Schwartz D, Bakker D, Fischl B, et al. Mechanisms of migraine aura revealed by functional MRI in human visual cortex. Proc Natl Acad Sci U S A 2001; 98: 4687-92.

Hartings JA, Andaluz N, Bullock MR, Hinzman JM, Mathern B, Pahl C, et al. Prognostic Value of Spreading Depolarizations in Patients With Severe Traumatic Brain Injury. JAMA Neurol 2020; 77: 489-99.

Kang EJ, Major S, Jorks D, Reiffurth C, Offenhauser N, Friedman A, et al. Blood-brain barrier opening to large molecules does not imply blood-brain barrier opening to small ions. Neurobiol Dis 2013; 52: 204-18.

Koroleva VI, Vinogradova LV, Bures J. Reduced incidence of cortical spreading depression in the course of pentylenetetrazol kindling in rats. Brain Res 1993; 608: 107-14.

Lehmenkuhler A, Sykova E, Svoboda J, Zilles K, Nicholson C. Extracellular space parameters in the rat neocortex and subcortical white matter during postnatal development determined by diffusion analysis. Neuroscience 1993; 55: 339-51.

Lehmenkühler A, Richter F, Poppelmann T. Hypoxia- and hypercapnia-induced DC potential shifts in rat at the scalp and the skull are opposite in polarity to those at the cerebral cortex. Neurosci Lett 1999; 270: 67-70.

Major S, Huo S, Lemale CL, Siebert E, Milakara D, Woitzik J, et al. Direct electrophysiological evidence that spreading depolarization-induced spreading depression is the pathophysiological correlate of the migraine aura and a review of the spreading depolarization continuum of acute neuronal mass injury. Geroscience 2020; 42: 57-80.

Maslarova A, Alam M, Reiffurth C, Lapilover E, Gorji A, Dreier JP. Chronically epileptic human and rat neocortex display a similar resistance against spreading depolarization in vitro. Stroke 2011; 42: 2917-22.

Mody I, Lambert JD, Heinemann U. Low extracellular magnesium induces epileptiform activity and spreading depression in rat hippocampal slices. J Neurophysiol 1987; 57: 869-88.

Olesen J, Larsen B, Lauritzen M. Focal hyperemia followed by spreading oligemia and impaired activation of rCBF in classic migraine. Ann Neurol 1981; 9: 344-52.

Oliveira-Ferreira AI, Milakara D, Alam M, Jorks D, Major S, Hartings JA, et al. Experimental and preliminary clinical evidence of an ischemic zone with prolonged negative DC shifts surrounded by a normally perfused tissue belt with persistent electrocorticographic depression. J Cereb Blood Flow Metab 2010; 30: 1504-19.

Pacheco JM, Hines-Lanham A, Stratton C, Mehos CJ, McCurdy KE, Pinkowski NJ, et al. Spreading Depolarizations Occur in Mild Traumatic Brain Injuries and Are Associated with Postinjury Behavior. eNeuro 2019; 6.

Revankar GS, Winkler MKL, Major S, Schoknecht K, Heinemann U, Woitzik J, et al. Spreading depolarizations and seizures in clinical subdural electrocorticographic recordings. In: Varelas PN, Claassen J, editors. Seizures in Critical Care A Guide to Diagnosis and Therapeutics. New York: Springer; 2017. p. 77-90.

Somjen GG. Mechanisms of spreading depression and hypoxic spreading depression-like depolarization. Physiol Rev 2001; 81: 1065-96.

Tomkins O, Friedman O, Ivens S, Reiffurth C, Major S, Dreier JP, et al. Blood-brain barrier disruption results in delayed functional and structural alterations in the rat neocortex. Neurobiol Dis 2007; 25: 367-77.

van Harreveld A, Stamm JS. Spreading cortical convulsions and depressions. J Neurophysiol 1953; 16: 352-66.

Voipio J, Tallgren P, Heinonen E, Vanhatalo S, Kaila K. Millivolt-scale DC shifts in the human scalp EEG: evidence for a nonneuronal generator. J Neurophysiol 2003; 89: 2208-14.

Woitzik J, Hecht N, Pinczolits A, Sandow N, Major S, Winkler MK, et al. Propagation of cortical spreading depolarization in the human cortex after malignant stroke. Neurology 2013; 80: 1095-102.
